# Sustainability on the Menu: Assessing the Role of Hospital Cafeteria Composting in Advancing Planetary Health Initiatives

**DOI:** 10.3390/ijerph23020146

**Published:** 2026-01-23

**Authors:** Lawrence Huang, Alex Jin, Katherine Wainwright, Joseph R. Junkin, Asghar Shah, Nadine Najah, Alexander Pralea, Bryce K. Perler

**Affiliations:** The Warren Alpert Medical School, Brown University, 222 Richmond Street, Providence, RI 02903, USAnadine_najah@brown.edu (N.N.);

**Keywords:** hospital food waste, composting, hospital sustainability initiatives, planetary health

## Abstract

**Highlights:**

**Public health relevance—How does this work relate to a public health issue?**

- The healthcare sector contributes an estimated 4.6% of global carbon emissions, and planetary health is a movement within the sector to address its emissions footprint.
- Supply chain-associated emissions contribute the largest proportion of healthcare’s footprint, with composting serving as a simple and scalable way for healthcare entities to address food waste.

**Public health significance—Why is this work of significance to public health?**

- This pilot-scale composting initiative diverted nearly 500 kg of food waste and prevented 0.35 metric tons of CO_2_-equivalent emissions over six months at an academic medical center cafeteria.
- By partnering with a community composting partner, cost parity with traditional waste disposal was calculated at a composting volume of 116 kg per day.

**Public health implications—What are the key implications or messages for practitioners, policy makers and/or researchers in public health?**

- Healthcare sustainability initiatives require integrated approaches combining infrastructure improvements, staff education, administrative support, and strategic partnerships with community organizations to overcome barriers of cost, contamination, and operational coordination.
- Composting programs can serve as both a waste reduction strategy and an educational avenue for engaging healthcare workers, patients, and administrators in planetary health concepts.

**Abstract:**

U.S. hospitals generate considerable food waste, contributing to environmental degradation strategies. This study evaluated the feasibility, impact, and perception of a novel composting program implemented at Rhode Island Hospital over six months beginning in December 2024. Compostable waste bins were installed in the cafeteria with educational signage. Surveys assessing composting knowledge, attitudes, and roles in waste management were distributed to staff, patients, and administrators. Collected food waste was transported to Bootstrap Compost, which provided daily weight data used to estimate greenhouse gas emissions reductions, compare composting with landfill disposal costs, and project annual outcomes. Over the study period, 490.6 kg of food waste were diverted from landfills, corresponding to a reduction of 0.35 metric tons of CO_2_-equivalent emissions. While composting was more expensive than landfill disposal ($6.45/kg vs. $0.24/kg), cost neutrality could be achieved with diversion rates at or above 116 kg per day. Surveys revealed strong support for composting but limited awareness of its relevance to healthcare’s environmental footprint. Respondents suggested improvements in education, signage, and infrastructure. This program demonstrated how hospital-based composting initiatives align with healthcare institutions’ environmental stewardship goals while highlighting financial and logistical challenges relevant for pilot–scale efforts.

## 1. Introduction

The growing field of planetary health focuses on the interrelated nature of human and environmental health [1]. The consequences of man-made greenhouse gas emissions and other pollutants have been linked to increased disease burden, whether from air pollution, extreme weather, infectious disease, falling crop yields, and mental health effects [1,2,3,4,5,6]. The World Health Organization (WHO) links an environmental risk factor to 23% of global deaths [7]. Conversely, the healthcare sector is a major source of carbon emissions, with estimates placing its contribution at 4.6% of the global total in 2021 [8]. The United States contributes the largest share of global healthcare emissions, while healthcare also comprises a disproportionately high 7.6% of its national emissions [9]. Altogether, the healthcare sector faces the dual responsibility of treating patients affected by climate change as well as mitigating its own footprint to prevent further harm.

Proposals for how the healthcare sector can contribute to broader adaptation and mitigation efforts include raising awareness of climate change’s health effects, supporting climate policy development, developing climate-resilient public health mechanisms, and contributing to financing flows [10]. The most direct action available to healthcare entities, particularly individual practitioners within systems, is to modify their own operations to reduce emission footprints [9,10]. A closer examination of this footprint reveals that 71% originates from supply chains, also known as Scope 3 emissions. Within this supply chain, agriculture contributes 9% to the global healthcare climate footprint, while waste treatment contributes an additional 3% [9]. As such, food waste represents a critical lever for healthcare organizations to reduce their impact.

This is especially true in nations with a high burden of food waste. The FDA reports that 30–40% of the American food supply is wasted annually [11]. This constitutes approximately 24.1% of municipal solid waste deposited in landfills [12]. A 2020 EPA report estimated that hospitals contribute 261,641 metric tons per year to this amount, with nursing homes contributing an additional 422,686 metric tons per year [13]. Individual facility studies find that a large part of food waste originates in food services. Specifically, the EPA report found waste generation factors of 296.26 kg of food wasted per hospital bed per year or up to 0.21 kg of food per meal [13]. One study conducted in a New York City hospital found that the hospital’s kitchen generated approximately 1.5 metric tons of solid waste daily, with the majority being sent to landfills [14]. This not only consumes limited landfill capacity but also generates greenhouse gases such as methane, hydrogen sulfide, and ammonia [15,16]. Of these gases, methane has a global warming potential 28 times higher than that of CO_2_ on a per-unit basis, while other gases are associated with negative human health effects [17].

Tackling food waste in healthcare has been addressed by initiatives ranging from international, top-down goal-setting to grassroots efforts. Prominent international and national campaigns include the WHO’s formation of the Alliance for Transformative Action on Climate and Health in 2022 and England’s National Health Service setting a goal of net zero by 2040 [18,19]. In the United States, the site of this study, Practice Greenhealth and Health Care Without Harm (HCWH) have been implementing sustainable healthcare delivery projects since 2001, with HCWH joining the U.S. Food Waste Pact in 2024 [20,21,22]. The U.S.-based Joint Commission, a non-profit responsible for accrediting over 20,000 providers, has also launched a voluntary Sustainable Healthcare certification aimed at reducing carbon emissions sources at participating entities [23]. Most of these efforts include food waste reduction as one solution among many to help decarbonize the sector.

**Figure 1:**
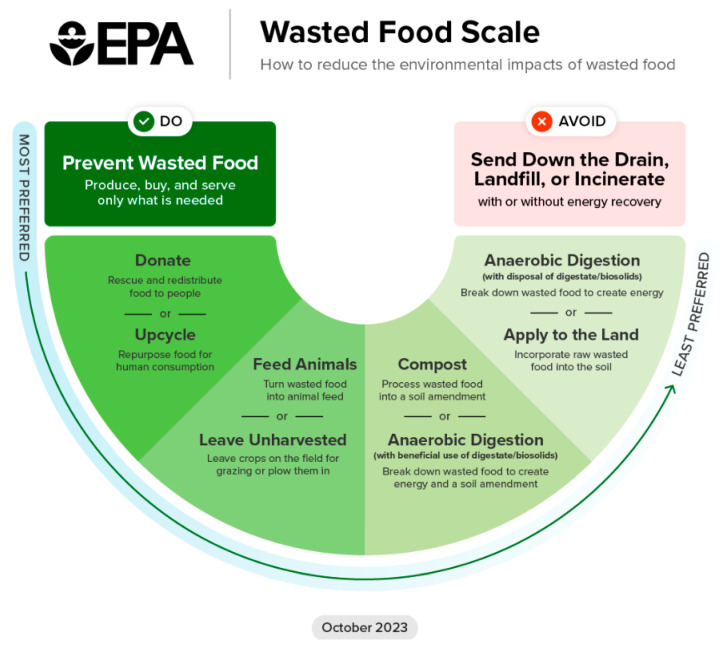
EPA Wasted Food Scale: How to reduce the environmental impacts of wasted food [24].

In contrast, the background literature documents numerous grassroots efforts exclusively focused on reducing hospital food waste internationally. The EPA Wasted Food Scale (Figure 1) provides a conceptual hierarchy with which to consider these projects, where waste prevention is favored over waste management strategies [24]. Prior studies have aimed to reduce waste by intervening on meal ordering services, meal design, and patient education [25,26,27]. Where prevention is not possible, the next priority is to divert waste from landfills. Common alternatives include donation, composting, and industrial uses, with less common options, including anaerobic digestion and feeding to animals [28,29,30]. Recent analysis indicates that food waste donation offers a significant 92% reduction in greenhouse gas emissions compared to landfill [28]. However, donations are frequently limited by food safety regulations, logistical constraints, and the reality that much hospital food waste is inedible and non-recoverable [28,29,31].

Composting yields the next-highest GHG reduction at 8.69% emissions reduction compared to landfill, outperforming industrial repurposing and anaerobic digestion [28]. It involves the aerobic breakdown of organic materials for intended use as soil enrichment, facilitating the return of nutrients to soil [29]. Environmental benefits include diverting food waste from landfills, reducing greenhouse gas emissions, and preserving water quality through reduced need for chemical fertilizers [32]. There are also concurrent economic benefits in the form of reduced waste-hauling fees and reductions in staff handling of waste [33]. However, successful composting initiatives require strong management support, organizational buy-in, proper education, and consistent follow-up to ensure ongoing effectiveness [34]. These factors are crucial to overcoming commonly encountered barriers, such as contamination of collected food waste, coordination issues across different departments, difficulties with staff training [30], waste odor [29], and challenges with evaluating real-time impact [33].

The evidence base surrounding hospital-based composting mostly consists of the gray literature, and only 13% of the included literature in a recent review focused on retail or commercial food waste within hospitals [30]. Within the United States, the authors are only aware of peer-reviewed studies investigating a single-day waste audit of a hospital kitchen, a multi-day integration of food waste collection across a hospital kitchen and cafeteria, and an initiative to repurpose hospital food waste for a community garden [35,36,37]. While the greenhouse gas reduction potential of composting has been quantified, widespread variability in measurement methods limits the ability to draw robust conclusions about operational viability [28]. Consequently, analyses comparing environmental benefits to implementation costs are needed to prioritize composting initiatives effectively [31].

This study contributes to the body of work by evaluating the acceptability, feasibility, and effectiveness of a pilot-scale composting program at Rhode Island Hospital (RIH). Developed collaboratively by hospital staff, medical students, and a community-based composting nonprofit, the initiative generated daily composting data for self-service compost bins in a hospital cafeteria setting. Waste diversion data enable further analysis of emissions mitigation and cost-effectiveness analyses. Surveys assessed sustainability attitudes across staff, patients, and administrators. These findings provide benchmarks for pilot-scale, community-partnered composting initiatives and illuminate the financial and behavioral challenges relevant to program expansion.

## 2. Materials and Methods

**Figure 2:**
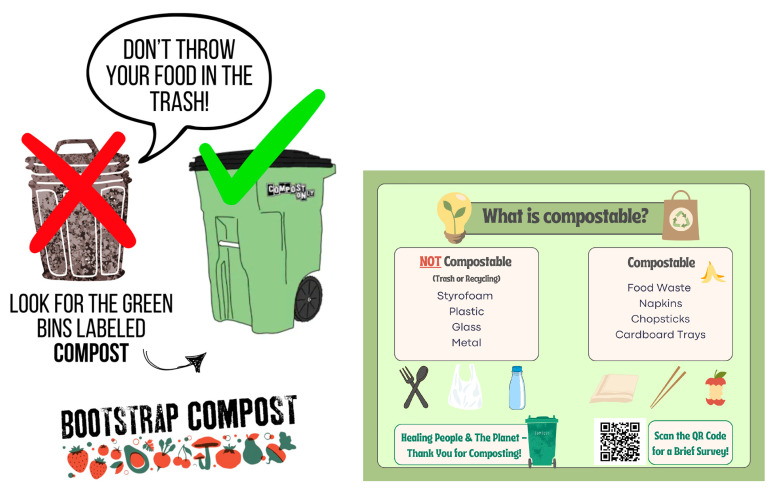
Instructional posters displayed in the hospital cafeteria.

This study evaluated a novel composting program at RIH over six months, starting in December 2024 through May 2025. The project was implemented in the hospital cafeteria in partnership with a regional composting company (Bootstrap Compost, Providence, RI 02904, USA) and was determined to be exempt from Institutional Review Board (IRB) oversight [38]. Patient consent was waived under 45 CFR 46.116 since no identifiable private information was collected. A 182 L composting collection bin provided by the company was placed alongside the main trash disposal bins in the hospital cafeteria. The bin, with an exchangeable biodegradable lining, was available to cafeteria users from 10 a.m. to 2 p.m. daily, Monday–Friday, to divert their organic waste. Clear instructional signage was created and displayed to guide users on appropriate waste separation and disposal (Figure 2).

In order to mitigate potential issues with overfilling, pests, and odor, the organic waste was transported by facilities staff from the cafeteria to the hospital loading dock daily. The waste was then collected daily from Monday to Friday by Bootstrap Compost and transported to regional farms, such as Rocky Hill Farm in Massachusetts, to be composted and converted into nutrient-rich soil. Bootstrap Compost charges a daily collection fee of $28, regardless of pickup weight. They also generated daily metrics on organic waste diversion, quantifying the mass of compostable materials redirected from landfill disposal. With the quantified daily organic waste diversion metrics provided by Bootstrap Compost, the EPA Waste Reduction Model (WARM) Version 16 was used to calculate the metric tons of carbon dioxide equivalent (MTCO_2_e) avoided by consequent landfill diversion [39]. The MTCO_2_e was then used to calculate environmental equivalencies utilizing the EPA Greenhouse Gas Equivalencies Calculator [40]. These calculations were performed by Bootstrap Compost using stopSuite software (version 1.4.16), which applies standardized parameters for typical composting scenarios within the EPA models [41]. To evaluate the overall financial performance of the program, we calculated the average cost per kilogram of composted material by dividing the total program cost by the total mass of compost collected over the study period. To contextualize this cost relative to conventional waste disposal, the program’s average cost per kilogram was compared conceptually with the hospital’s fixed landfill disposal fee. A cost-parity and capacity analysis was conducted using the fixed $28 daily fee to determine the composted mass needed for the composting program to match the landfill disposal rate.

To assess perceptions of the program, the research team developed an electronic survey targeting hospital staff, patients, medical providers, and visitors in the cafeteria. Participants were recruited both through in-person engagement by volunteers stationed near the composting bin and via a QR code displayed on nearby signage, allowing individuals to complete the survey independently even when volunteers were not present. Due to this collection methodology, the response rate could not be assessed as daily cafeteria user counts vary. The survey recorded the respondents’ job functions, prior knowledge of food waste issues and attitudes toward composting in healthcare environments. Questions were a combination of Likert survey questions used to measure familiarity with composting, the environmental impact of hospital food waste, and the U.S. healthcare system’s carbon footprint, as well as open-ended responses for suggestions related to composting and reducing environmental impact. Likert-scale responses were used as indicators of participants’ knowledge of healthcare’s environmental footprint and support of the composting initiative. Open-ended responses were thematically coded to identify common perspectives, barriers, and suggestions for program improvement.

## 3. Results

### 3.1. Waste Diversion and Carbon Emissions 

**Figure 3:**
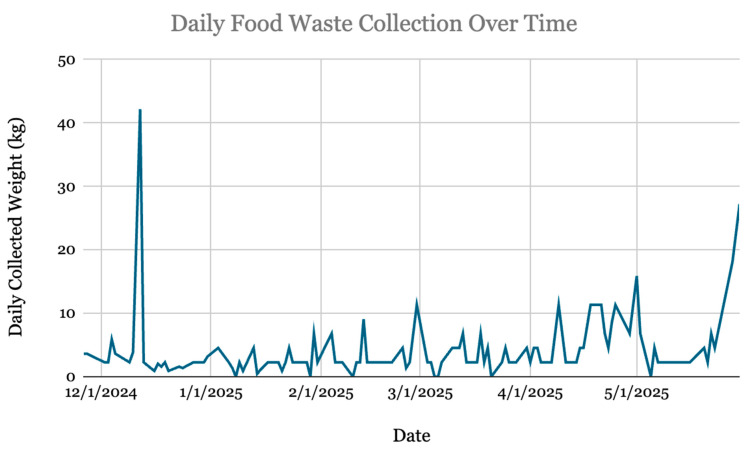
Daily weights (kg) of compost waste collected over study period.

**Figure 4:**
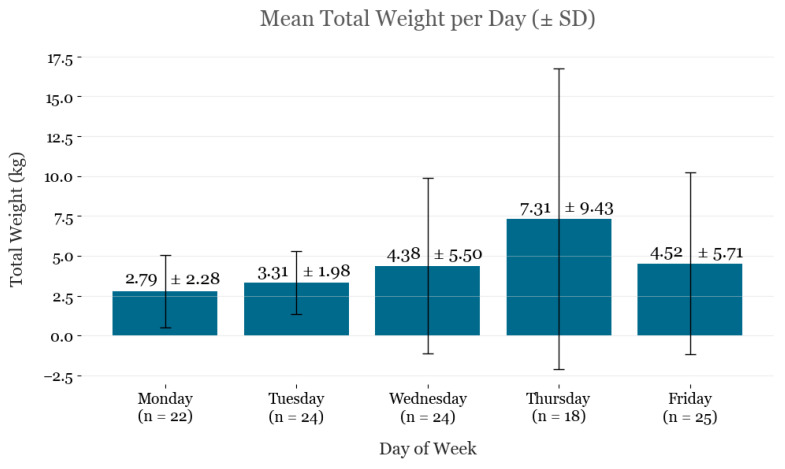
Mean compost collected over the study period, stratified by weekday.

After a six-month period of compost collection, a total of 490.6 kg of food waste was diverted from landfills. Compost was scheduled for collection for 113 days during this study period, of which 0 kg of waste was collected on seven days. Across those 113 days, an average of 4.4 kg of food waste was collected and diverted to composting daily. There were a total of 21 days with 6.8 kg or more of food waste collected, and the most food waste collected on a single day was 42.2 kg. Figure 3 visualizes the temporal trend in collected daily waste. Of note, the day with the most waste collection (Thursday, 12 December 2024) was also the day when volunteers were present in the cafeteria for 3 h to direct users toward the composting bin. Figure 4 stratifies the mean collected waste by day of the week. Collection volumes remained relatively consistent on Mondays (2.79 ± 2.28 kg) and Tuesdays (3.31 ± 1.98 kg), with an apparent increase later in the week, peaking on Thursday (7.31 ± 9.43 kg). However, the larger standard deviations observed from Wednesday through Friday indicate that outlier values on these days inflated the mean collection weights. The total waste diverted to composting resulted in a reduction of 0.35 MTCO_2_e. This is equivalent to 149.1 L of gasoline consumed.

### 3.2. Cost Analysis

The majority of costs associated with the composting program were incurred due to compost collection services provided by Bootstrap Compost. Over the 6-month study period, compost was collected on 113 days at a daily pickup cost of $28, resulting in a total cost of $3164. With 490.6 kg of compost collected, the average cost per kilogram was $6.45. The lowest daily cost achieved was $0.66 per kg when 42 kg of food waste was composted. In comparison, RIH’s open container waste disposal fee is $0.24 per kg.

At a $28 daily pickup rate, 116 kg of composted waste per day would result in a cost equilibrium to the conventional waste disposal cost of $0.24 per kg. Using assumptions of food waste density averaging between 0.5 and 1 kg per liter, the 182 L collection bins could capture 91 to 182 kg of food waste daily [42]. This demonstrates that extensive usage of just one composting bin could be sufficient to achieve cost parity. At the upper limit of food density (1.0 kg per liter) with full utilization of the 182-L collection bin capacity, the composting cost would be $0.16 per kg ($28 ÷ 182 kg). Assuming 20 pickup days per month at maximum capacity, this would represent an annual cost savings of 240 days × 182 kg × $0.08 savings per kg = $3494 compared to conventional waste disposal. Additionally, Bootstrap Compost provides $132 worth of complimentary composting credits annually, bringing the total annual savings to $3626. Even at the middle range of food density (0.75 kg per liter), with similar assumptions as above, the total annual savings still amount to $1115. At the lowest estimated food density (0.5 kg per liter), this program would result in a net $1478 loss.

### 3.3. Survey Responses

During the same six months of compost collection, a total of 45 survey responses were collected; most were healthcare workers or other clinical staff (35/45). Using a 5-point Likert scale, ratings of 3/5 or lower were classified as indicating low familiarity. Ratings averaged 3.07 (SD = 1.32) for composting, 2.44 (SD = 1.24) for the carbon footprint of the U.S. healthcare system, and 2.20 (SD = 1.34) for food waste generated by U.S. hospitals, revealing significant knowledge gaps across all three surveyed topics (Table 1). While composting represented the most familiar concept, 60% of respondents (27/45) still demonstrated low familiarity. Knowledge of healthcare-specific environmental impacts proved even more limited. Approximately 73% of participants (33/45) reported low familiarity with the U.S. healthcare system’s carbon footprint. Most notably, 78% of respondents (35/45) indicated low familiarity with hospital food waste generation, representing the area of lowest awareness.

In contrast, when asked about the importance of diverting hospital food waste from landfills, the majority (23/45) responded that waste diversion is “very important” with a mean score of 4.13 (SD = 1.32) (Table 1). All but three respondents (42/45) think this program would benefit from informational posters/signage near the composting bins that explain what items can be composted.

Respondents were also asked to suggest initiatives for further food waste reduction in the hospital setting (Table 2). Of the 45 surveys completed, 26 respondents provided open-ended comments, which generated 27 unique coded themes. The most frequently mentioned theme was education and awareness (26%), one respondent suggesting “offer compost education at the cafeteria monthly.” Increased signage was also mentioned (11%), which could support and reinforce educational initiatives. Other common themes included infrastructure and institutional support (15%), such as expanding composting bin availability; food systems changes, including sustainable ingredients and packaging (15%); limiting portion sizes (15%); and better-tasting food options (11%), with one participant recommending to “have more affordable non processed and prepackaged items in the cafeteria.” These suggestions were aligned with the EPA food waste reduction, recovery and recycling hierarchy [24]. Overall, prevention of food waste was most frequently mentioned, although food donations were also often suggested by respondents. One notable example included allowing “cafeterias to donate leftover food to staff or visitors. Perhaps form a connection with homeless shelters in the area for pick up.”

## 4. Discussion

Composting offers a practical and scalable solution for healthcare organizations to engage with planetary health goals. This project report documents the successes and challenges of a novel composting implementation at a large academic hospital in the United States. During the six-month study period, a limited composting pilot achieved an average daily diversion of 4.4 kg of food waste, equivalent to 0.35 MTCO_2_e of avoided carbon emissions. This was accomplished by a small team of volunteers partnering with hospital facilities staff and an outside composting vendor, demonstrating the relatively low barrier to implementation. The only substantial in-hospital infrastructure needed was a composting toter, waste collection bags, and printed educational materials. The composting vendor provided built-in data-collection capabilities that enabled daily waste tracking and emissions-reduction calculations. This last capability is increasingly essential for hospitals to meet reporting requirements for initiatives such as the Joint Commission’s Sustainable Healthcare Certification [23].

Composting was also found to be an opportunity for sustainability teams to engage in education and feedback. This was best achieved through the visible placement of the composting toter as well as volunteer-administered surveys. Among survey respondents, most demonstrated greater familiarity with general sustainability concepts than with healthcare-specific environmental challenges. Despite this knowledge gap, a majority of respondents advocated for the diversion of food waste from U.S. hospitals, suggesting support for sustainability initiatives even when the connections between healthcare and environmental crises are not fully understood. This is consistent with national data showing that many hospitals express interest in composting or anaerobic digestion; however, very few actually implement such systems [43]. Respondents recognized that more initiatives need to be emphasized to improve hospital sustainability efforts and expressed that improved educational materials and signage would help further reduce hospital-generated food waste. Notably, this self-reported survey data is subject to response bias, and a larger, more diverse sample is needed, especially given the majority representation of healthcare workers.

Our limited study has several additional limitations. Firstly, the study was performed in a single hospital over six months, which limits generalizability to other healthcare facilities. The parameters used for emissions calculations are also proprietary to the analytics vendor utilized by Bootstrap Compost. Additionally, total waste data is not yet available for our hospital, but an academic hospital in New York City with a similar number of beds estimated that its kitchen produces around 1500 kg of food waste daily [14]. The daily waste collected by a cafeteria composting toter can capture only a small fraction of this total due to a single site of collection, limited volunteer staffing to encourage composting, and larger waste streams in other parts of the food-handling process. In contrast, the previous literature achieving higher collection volumes required full-time staff support and integration into various workflow steps across both the kitchen and the cafeteria [37]. This indicates a tradeoff between waste collection at scale and ease of implementation. This is further illustrated by the study’s peak collection day, which coincided with volunteer-directed waste sorting. This finding suggests that collection volumes are partially constrained by user participation rather than waste availability. More consistent staffing support could substantially increase composting diversion rates.

The economic analysis also revealed substantial cost challenges that must be addressed for program sustainability. The cost to implement this program averaged $6.45 per kg of diverted waste, with the most efficient day achieving $0.66 per kg. The current cost structure is significantly more expensive per unit weight than the hospital’s standard open-container waste disposal fee. However, the benefit of a fixed-cost compost pickup service is that cost savings can be achieved with scale, with a cost equivalence occurring at 116 kg of food waste diverted daily. This threshold is well within the capacity of the provided 182 L collection bins and could lead to annual cost savings of $3494 per collection bin at maximal capacity. However, this still represents a 26-fold increase in currently observed collection volumes, indicating that additional scale will be necessary to meet cost parity. Our findings align with previous research on small-scale composting initiatives in institutional settings. For example, composting efforts at the University of Kentucky and the University of Minnesota–Morris reported substantial environmental benefits and widespread stakeholder support but also noted significant financial and operational challenges [44,45].

These results underscore the critical importance of operational efficiency in composting program implementation. Specifically, within healthcare institutional settings and in collaboration with community-based organizations, optimizations are essential regarding collection frequency protocols and the physical placement of composting infrastructure. Additional savings are also possible through volume discounts offered by partnered community composting programs. Maximizing organic waste diversion volumes while minimizing collection frequency would reduce per-unit operational expenditures. However, the determination of these operational parameters is contingent upon facility-specific and partnership-dependent logistical considerations. Critical variables include user accessibility to collection points, temporal fluctuations in waste generation patterns, integration with existing waste management systems and personnel resources, and operational flexibility from partnering community organizations. Continuous data collection and monitoring of cost effectiveness would be essential for achieving long-term program sustainability and securing sustained administrative support.

To address previous limitations, an expansion and re-imagining of this initiative will be required. Previously, compost collection bins were only placed in the cafeteria for four hours daily, Monday to Friday, and not at other potential food waste sites across the hospital, such as staff break rooms. Indeed, this was suggested by some survey participants in free-response questions. The mobile compost collection bins were also not in a fixed location, periodically being moved to different locations in the cafeteria by hospital staff. When this movement occurred, the bins were in locations where there was no clear signage, which limited visibility of these efforts. As for the food waste collection itself, there is potential for contamination or sorting inaccuracy, which may influence monitoring of waste tracking by weight. As discussed earlier, balancing the expansion of collection while maintaining high collection volumes per bin is critical for maintaining sustainable per-unit composting costs. The development of built-in composting stations with 24-h accessibility at waste sorting stations, combined with consistent staffing and clear signage, would create more effective compost collection. Creating a compost champion role presents a unique opportunity for healthcare staff and trainees to get involved in optimizing waste sorting and diversion. Healthcare systems can partner with medical schools to establish volunteer systems for this champion role. This project was also recently granted approval from the Warren Alpert Medical School of Brown University for volunteer hours with our composting to fulfill required service learning hours. Expanding to multiple hospitals in Rhode Island could enhance results and is an important future direction.

As noted by survey respondents, educational improvements are essential for program success. Various platforms can be utilized, from onboarding with asynchronous modules about composting efforts to lecture series and Grand Round presentations. Signage should be clearly marked at compost bins with pictorial representations of waste streams that can be diverted. Multiple fixed locations for compost bins with clear, large signage will help draw attention to these efforts and improve waste sorting at disposal stations. Establishing connections between the large carbon footprint of hospitals and the health harms of climate change may encourage greater participation in sustainability initiatives. Future research could also include waste audits to assess participant compliance with educational composting flyers and true composting effectiveness.

## 5. Conclusions

This hospital-based composting initiative demonstrated that food waste diversion is both feasible and impactful, with a limited pilot aimed at general cafeteria users diverting nearly 500 kg of food waste over six months and preventing an estimated 0.35 MTCO_2_e. This result demonstrates the impact that a simple composting implementation can achieve while also highlighting the critical challenges that persist at this scale. While the average cost of composting significantly exceeded landfill disposal, modeling showed that cost neutrality is achievable at higher diversion volumes. Achieving such targets and meaningful cost savings for hospital systems will require increased integration into current waste flows and expansion of collection sites, as well as access to volume-based discounts from community composting partners. The next phase of this project aims to implement these improvements.

Among survey participants, responses revealed strong support for food waste diversion but identified limited awareness of healthcare-specific sustainability challenges. Respondents emphasized the need for improved education, signage, and infrastructure, such as fixed, clearly marked compost bins. Additional study is required to better characterize attitudes amongst a broader base of users, not just voluntary respondents. Beyond collecting responses, the visibility created through survey administration and volunteer engagement demonstrates how a composting program can function as a platform for sustainability education, facilitating dialogue between students, staff, patients, and community partners. Initial data collection at this scale, both quantitative and qualitative, is critical for engaging wider hospital leadership, continuing end-user education, and integrating into existing operational workflows. These grassroots efforts will be necessary as one component of increasingly organized institutional efforts to address healthcare’s environmental footprint.

It is now more important than ever that sustainability efforts to reduce food waste be broadly implemented, not only to improve human health but also to allow our planet to heal. The scope of this problem is formidable, and no single strategy will be sufficient to sustainably manage our food waste, but composting can serve as both a practical waste reduction strategy and a catalyst for broader environmental stewardship within healthcare systems.

## Figures and Tables

**Table 1:** Survey Respondent Familiarity of Environmental Topics and Perceived Importance of Food Waste Diversion (*n* = 44–45).

Survey Item	Mean (SD)	% ≤ 3	% > 3	*n*
How familiar are you with composting?	3.07 (1.32)	62.2%	37.8%	45
How familiar are you with the carbon footprint of the U.S. healthcare system?	2.44 (1.24)	73.3%	26.7%	45
How familiar are you with the food waste generated by U.S. hospitals?	2.20 (1.34)	81.8%	18.2%	44
How important is it to divert food waste generated by U.S. hospitals away from landfills?	4.13 (1.32)	24.4%	75.6%	45

**Table 2:** Themes Identified from Open-Ended Responses on Food Waste Reduction Initiatives (*n* = 26).

Theme	*n* (%)	Representative Quotes
Education and Awareness	7 (26%)	“Offer compost education at the cafeteria monthly.”
Infrastructure and Institutional Support	4 (15%)	“More composting bins.”; “C-suite buy-in”
Sustainable Ingredients and Packaging	4 (15%)	“Using reusable utensils instead of plastic.”
Portion Control	4 (15%)	“Limiting portion sizes.”; “Smaller portions.”
Improved Signage	3 (11%)	“More posters around the hospital.”; “Clear signs.”
Better Tasting Food	3 (11%)	“Better food.”; “Non-processed.”
Food Donation and Redistribution	2 (7%)	“Allow cafeterias to donate leftover foods.”

## Data Availability

Data supporting the findings of this study are available from the corresponding author upon reasonable request.
